# The Impact of the COVID-19 Pandemic on Forensic Mental Health Services and Clinical Outcomes: A Longitudinal Study

**DOI:** 10.3389/fpsyt.2021.780236

**Published:** 2022-01-18

**Authors:** Ignazio Puzzo, Luke Aldridge-Waddon, Nicholas Stokes, Jordan Rainbird, Veena Kumari

**Affiliations:** ^1^Department of Life Sciences, Centre for Cognitive Neuroscience, Brunel University London, Uxbridge, United Kingdom; ^2^West London Forensic Service, West London National Health Service (NHS) Trust, Southall, United Kingdom

**Keywords:** forensic psychiatry, COVID-19, forensic mental health service, inpatients, clinical outcomes

## Abstract

**Background:**

The COVID-19 pandemic has had a substantial impact on forensic mental health service provision and implementation. This study aimed to provide an analysis of the impact of COVID-19 related restrictions on routine outcomes within a large forensic mental health service in London, UK.

**Method:**

We conducted a longitudinal cohort study using data collected routinely prior to the COVID-19 pandemic (April 2018–March 2020) and then stages thereafter (March 2020–March 2021; analyzed as March–May 2020, June–September 2020, October–December 2020, January–March 2021). We used causal impact models (Bayesian structural time-series) to examine the effect of COVID-19 related changes on routine outcomes related to service provision and implementation.

**Results:**

There was an overall increase in long-term segregation (LTS) hours during the pandemic; 140%, (95% Cl 107, 171%) during Lockdown 1; 113%, (159% Cl 127, 192%) during post-Lockdown 1; 45% (95% Cl 23, 68%) during Lockdown 2 and, finally, 90% (95% Cl 63, 113%) during Lockdown 3. The most negative outcomes were evident during Lockdown 3. Incidents of violence were significantly more frequent during Lockdown 3 than would have been predicted based on pre-pandemic data, including physical assaults to service users (206%, 95% CI 57%, 346%), non-physical assaults to service users (206%, 95% CI 53%, 339%), and self-harm (71%, 95% CI 0.4%, 135%). Use of enforced medication also increased during Lockdown 3 (317%, 95% CI 175%, 456%).

**Conclusion:**

The pandemic and its related restrictions negatively affected some service outcomes. This resulted in increased incidents of violence and increased use of restrictive interventions, beyond what would have been expected had the pandemic not occurred.

## Introduction

Inpatient forensic mental health services provide specialist psychiatric care and treatment within conditions of high, medium, and low security. Most individuals receiving care within these settings present with a complex mental health history and demonstrate a level of risk to others (and to themselves) which requires treatment within a secure environment.

The COVID-19 pandemic has had a substantial impact on forensic service practice (1, 2). National restrictions (3), in combination with pressure on national health services, resulted in changes in service provision and implementation, including changes in staff working patterns, physical distancing, telemedicine, admission and discharge procedures, and COVID-19 case detection and isolation (4). A scoping review of the literature examining the impact of COVID-19 in the prison population (5) suggested that imprisoned individuals were: (i) at a higher risk (compared to the normative population) of contracting COVID-19, related to living in confined spaces (overcrowded, poorly ventilated and often insanitary environments) and poorer physical heath; (ii) severely impacted by infection prevention and control measures which restricted access to each other and outside visitors, resulting in more isolation than normally experienced. The authors concluded that these conditions posed a huge challenge to the mental health of prisoners and staff working within prisons, and thus identified understanding the impact of COVID-19 and related restrictions as an urgent need (5).

Like the impact of COVID-19 within the prison system, changes associated with the pandemic have added to the challenges already faced by forensic mental health services (6). Indeed, recent commentaries [e.g., (6–10)] describe immediate and long-term impacts of the pandemic on service delivery and clinical decision-making, as well as negative effects on staff and service user psychological wellbeing.

Forensic mental health service users are highly vulnerable to adverse COVID-19 outcomes due to risk factors such as obesity, hypertension, metabolic syndrome, and neurocognitive impairments (11), and the nature of these services means that positive cases can spread quickly if not isolated (12). Almost one-third of service users within a forensic mental health service meet high risk age criteria, perhaps suggesting a greater than expected risk of adverse outcomes in the event COVID-19 infection (11).

In addition to these physical health vulnerabilities, the COVID-19 pandemic has had a marked effect on the mental health of those with pre-existing mental health difficulties, increasing feelings of health-related anxiety, stress, depression (13), and consequently Pozza et al. (14) propose that the stressors associated with the pandemic (and subsequent changes in forensic service delivery) will have exacerbated the psychopathological symptoms of some forensic mental health service users.

Routine outcomes are used to assess effectiveness within inpatient forensic services (15). At a service level, outcome data include length of stay (16), discharge rates (17), movement between levels of security (15), frequency of inpatient violence (18), and incidents of disruptive behavior (19). At a service user level, risk assessments, such as the HCR-20v3, are used to index treatment progression (15). Forensic mental health services have implemented service-wide changes in practice in response to the pandemic. Systematic reviews [e.g., (20)] and various commentaries (6, 9, 10) have described how the COVID-19 pandemic has impacted psychiatric and mental health facilities and professionals globally. However, there is no empirical work to-date examining how these changes have affected forensic service outcomes, including frequency of incidents of violence, restrictive interventions, and service user level of risk.

The objective of the present study is, therefore, to provide an analysis of the impact of COVID-19 related restrictions on routine outcomes within a forensic mental health service in London, UK. This study aims to characterize how the three UK national lockdowns impacted service implementation and outcomes, and thereby support clinicians and policymakers in understanding how the pandemic has affected service user experience. The presented results may also inform service delivery, should similar emergencies occur in the future.

## Methods

### Description of Forensic Mental Health Service

The inpatient service has 246 beds and offers treatment and rehabilitation across medium and low secure settings for adult men and women. Service users typically have complex needs related to severe and enduring mental disorders (mainly psychotic disorders and/or personality disorders related difficulties) and offending behavior, and many have significant histories of substance use. Service users are formally admitted and detained under the Mental Health Act 1983 (amended 2007), with a mean length of stay of 3.5 years.

**Figure 1:**
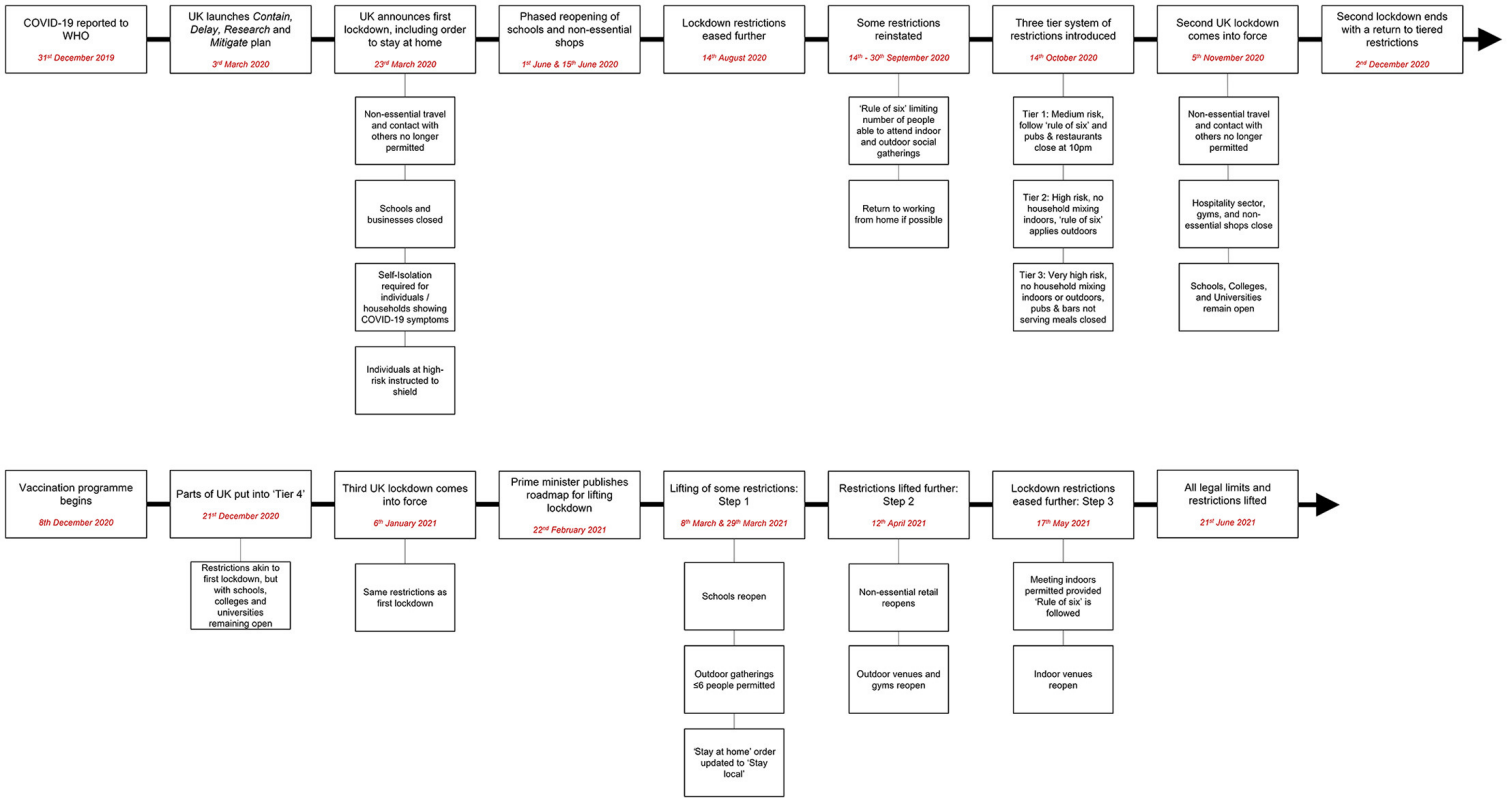
COVID-19 pandemic in the UK.

Changes in service provision and implementation in response to the pandemic were coordinated by a COVID-19 clinical governance committee and considered in other clinical governance forums, including a Restrictive Interventions Monitoring and Review Group. All changes were reviewed daily initially and at least weekly throughout the pandemic in-line with legal requirements and government guidance (21). Some specific decisions were also subject to additional review by a Trust-wide COVID Clinical Ethics Group created to examine the ethics of complex COVID-19 related decisions. A flowchart of key COVID-19 events and timepoints in the UK is presented in Figure 1.

### Routine Outcome Data Extraction

This study included a range of outcome data collected routinely within the service. Data were accessed for the period 1st April 2018 to 5th April 2021. Descriptions of the routine outcome data that were accessed are provided in Table 1.

**Table 1:** Extracted routine outcome data.

	**Extracted**	**Description**
**Service level**		
**Outcome type**		
**Incidents of violence**		
Physical assaults to staff	Weekly frequency, beginning 1st April 2018	Defined as attempted and actual physical assaults by service users to staff, such as punching, pushing, head-butting
Physical assaults to service users	″	Defined as attempted and actual physical assaults by/to service users, such as punching, pushing, headbutting
Non-physical assaults to staff	″	Defined as verbal assaults by service users toward staff, such offensive remarks or threats
Non-physical assaults to service users	″	Defined as verbal assaults by/to service users, such as offensive remarks or threats
Self-harm	″	Defined as attempted and actual incidents of self-harm, such as cutting, inserting or use of ligature
Damage to property	″	Defined as hitting, destroying, or breaking either own or hospital's property / structures
**Restrictive interventions**		
Short-term seclusion (STS) hours	weekly total hours, beginning 1st April 2018	Supervised confinement and isolation of a service user, normally in a seclusion room, in order to contain severely disturbed behavior that is likely to cause harm to others
Long-term segregation (LTS) hours	″	Longer term confinement of a service user in order to manage sustained risk to others which is a constant feature of the service user's presentation, usually commenced after a period of 7 days in STS
Enforced medication	Weekly frequency, beginning 1st April 2018	The enforced administration of injectable psychotropic medication, usually due to a service user repeatedly refusing their regular medication or the need to rapidly calm an agitated service user
Continuous therapeutic engagement and supportive observation (TESO) hours	″	The service user is always kept within eyesight of one member of staff due to increased risks. Depending on risk, may also involve observation by two or more members of staff, with one being within arm's length
Intermittent TESO hours	″	The service user is observed and therapeutically engaged at a higher than usual frequency, normally every 15 minutes, due to increased risks
**Care pathway progression**		
Admissions	Weekly frequency, beginning 1st April 2018	Defined as entry into forensic service
Discharges	″	Defined as exit from forensic service to other hospital service, prison, or community
**Patient level**		
HCR-20v3		Structured risk assessment tool typically completed by a trained clinician every 6 months, includes items pertaining to historical factors, clinical factors, and risk factors^*^
Clinical factor	– Most recent prior to 7th March 2020 – Between 8th March 2020 and 30th September 2020 – Between 1st October 2020 and 31st March 2021	Recent psychosocial adjustment which captures relatively short-term changes. Includes 5 items: difficulties with insight, violent ideation or intent, symptoms of major mental disorder, instability, and treatment or supervision response
Risk factor	″	Anticipated psychosocial adjustment, based on goals and plans for the future. Includes 5 items: problems with professional services and plans, living situation, personal support, treatment or supervision response, and stress and coping

* *Only clinical and risk factors are examined because they change dynamically over time; Historical is a static risk factor and thus would not show any change over time*.

### Routine Outcome Data Processing

Weekly outcome data were extracted from 1st April 2018 onwards, with the period up to and including 7th March 2020 defined as pre-pandemic. The data gathered during the COVID-19 pandemic were divided into smaller segments to dynamically reflect the three UK national lockdowns (21). These included the following time windows: 12 weeks during Lockdown 1 (8th March 2020 to 31st May 2020); 16 weeks post-Lockdown 1 (1st June 2020 to 30th September 2020); 12 weeks during Lockdown 2 (1st October 2020 to 31st December 2020); and 12 weeks during Lockdown 3 (1st January 2021 to 31st March 2021). The routine outcome data were thus organized as pre-pandemic, Lockdown 1, post-Lockdown 1, Lockdown 2, and Lockdown 3.

Whilst the 2nd UK national Lockdown officially ran from 5th November to 2nd December 2020, London was subject to high levels of restriction before and after these dates under the national “tier” system, so the whole of the period 1st October 2020 to 31st December 2020 is referred to as Lockdown 2 for this analysis.

### Data Analysis

**Figure 2:**
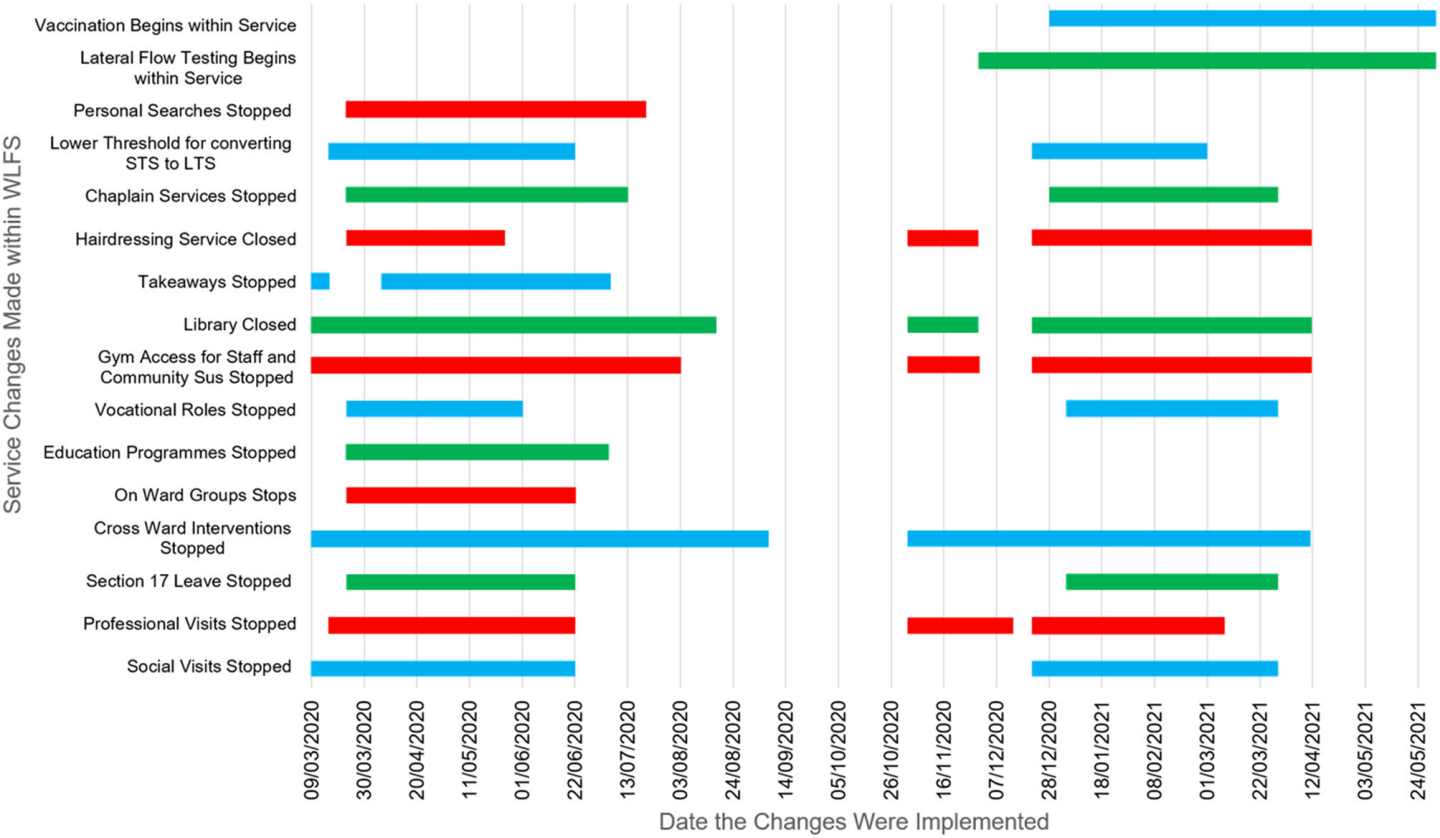
Gantt chart of changes in forensic mental health service implementation.

**Figure 3:**
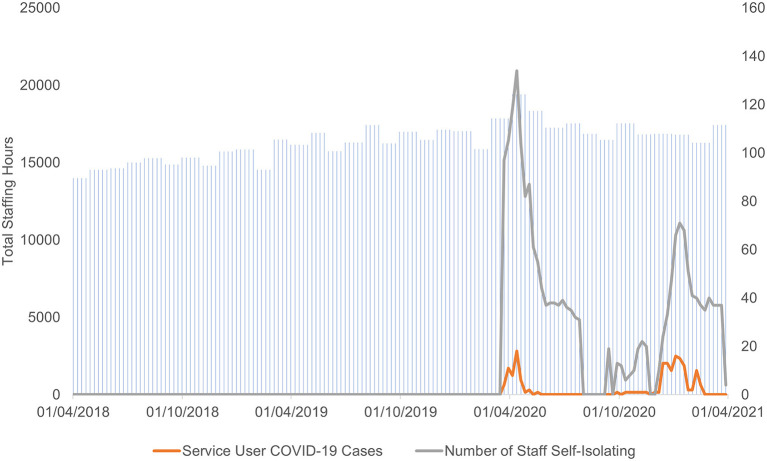
Plot of weekly COVID-19 cases against staff self-isolation and total staffing hours for the entire service.

There were three stages to the analyses. First, changes in practice in response to the pandemic were plotted (Figure 2) and analyzed descriptively. To estimate prevalence of the virus and the impact of self-isolation within the service, COVID-19 cases were plotted against the number of staff self-isolating and total staffing hours (Figure 3).

Second, we ran a series of causal impact models [which apply Bayesian structural time-series (22)] to infer and examine the effect of the COVID-19 related changes in service provision and implementation on incidents of violence, restrictive interventions, and care pathway progression. Causal impact models infer the effect of an event (e.g., COVID-19 pandemic related restrictions) on an outcome (e.g., incidents of violence) by estimating what would have happened to the outcome if that event did not occur, and then compare that with what happened to the actual outcome following the event. The difference between the predicted outcome (e.g., incidents of violence) in the absence of the event (no COVID-19 pandemic related restrictions) and the observed outcome after the event is the estimation of the impact of the event on the outcome.

To control for confounding variables that might partially explain change in routine outcomes, weekly total staffing hours and seasonality were included as covariates (with seasonality entered as nine 12-week seasons). The models were run using the Causal Impact Package that is available on CRAN (https://cran.r-project.org/web/packages/CausalImpact/vignettes/CausalImpact.html).

Third, changes in HCR-20v3 scores across time were assessed using the Friedman test (23). HCR-20v3 scores from a sample of 137 service users (111 males and 26 females) were compared at three timepoints: (i) pre-pandemic, (ii) during the first 6 months of the pandemic, and (iii) during the second 6 months of the pandemic. Item and total scores for both Clinical and Risk scales were analyzed.

### Ethics

This study was performed in accordance with the Declaration of Helsinki. It was approved as a service evaluation by the Clinical Effectiveness and Audit Department (West London NHS Trust) and the Research Ethics Committee Brunel University London−27418-LR-Dec/2020- 29482-1. Informed consent was not required as the data used in this study were fully anonymised clinical routinely collected data.

## Results

### Characterizing Impact of COVID-19 Pandemic on Service Implementation

The COVID-19 pandemic and subsequent restrictions markedly affected service provision and implementation. As shown in Figure 2, social visits were stopped at intervals during the pandemic, and only reinstated in-line with government guidance. Professional visits were reduced or stopped at intervals, with in-person visits from legal representatives only permitted prior to court cases or tribunal hearings. As an alternative, professional meetings and social contact (family and friends) were facilitated virtually where possible. Mixing of service users between wards (off-ward therapy and recreational areas) was largely stopped throughout most of the pandemic, with each ward constituting a “bubble” kept separate from other wards.

Service users are ordinarily able to access planned leave from the hospital (Section 17 leave) when authorized by their responsible clinician, and when required by the Ministry of Justice. Section 17 leave is important for rehabilitation and includes leave to local and residential areas, as well as overnight leave to hostels prior to discharge. Section 17 leave was stopped or limited on two occasions (for ~3 months each time) across the course of the pandemic (Figure 2) following government guidelines.

Therapeutic group interventions which involved individuals from different wards were stopped in the week commencing 9th March 2020 and, aside from a brief interlude in September and October 2020, did not gradually resume until April 2021. These were replaced by individual sessions or ward-based groups. In line with national restrictions in the wider community, access to libraries, hairdressers, religious services, gyms, educational activities, and vocational roles, was also reduced, stopped, or provided on-ward.

Finally, care plan approach and HCR-20v3 review meetings were initially suspended, except in the instance of admission, discharge, transfer, or in the event of a serious incident. A policy was also introduced to reduce the threshold for converting short-term seclusion (STS) to long-term segregation (LTS), to decrease the need for frequent medical and multidisciplinary reviews, thereby reducing transmission risk. Some security measures were adjusted to assist with infection control; for example, “pat down” searches were suspended and then resumed on an intelligence-led basis.

### Impact of COVID-19 Pandemic on Routine Outcome Data

#### Incidents of Violence

Incidents of violence included 6 categories: physical assault to staff, physical assaults to service users, non-physical (verbal) assaults to staff, non-physical assaults to service users, self-harm, and damage to property (Table 1). The model for physical assaults to staff demonstrated an overall reduction during the pandemic, showing a statistically significant 53% reduction during Lockdown 2 in comparison to prediction. On the contrary, the model estimating physical assaults to service users indicated an overall increase which reached a significant effect (206% increase) during Lockdown 3 (Table 2A) and a 93% increase post-Lockdown 1. The model estimating non-physical assaults to staff revealed a mixed picture, with a significant 36% decrease during Lockdown 1 and a significant 52% increase vs. prediction post-Lockdown 1. Non-physical assaults to service users revealed a significant 206% increase during Lockdown 3 and a 93% increase post-Lockdown 1 (Table 2A). Similarly, the model on self-harm revealed a significant 71% increase during Lockdown 3 compared to what would have been expected based on prediction. No significant changes in incidents of damage to property were observed across the pandemic (Table 2A).

**Table 2:** Output from models predicting routine outcome using pre-pandemic data.

		**Actual**	**Prediction**	**95% CI**	**Absolute**	**95% CI**	**Relative**	**95% CI**	***p***	**↑↓**
			**(sd)**		**Effect (sd)**		**Effect (sd)**			
**A. Incidents of violence**
Physical assault to staff	Lockdown 1	3.5	5.4 (1.6)	[2.4, 8.8]	−2 (1.6)	[−5.3, 1.1]	−36% (29%)	[−98%, 20%]	0.102	↓^ns^
	Post-Lockdown 1	3.2	4 (1.2)	[1.7, 6.7]	−0.83 (1.2)	[−3.5, 1.5]	−21% (31%)	[−87%, 37%]	0.243	↓^ns^
	Lockdown 2	2.8	5.9 (1.3)	[3.5, 8.6]	−3.1 (1.3)	[−5.8, −0.74]	−53% (22%)	[−98%, −13%]	**0.009**	↓^*^
	Lockdown 3	3.1	4.6 (1.2)	[2.4, 7]	−1.5 (1.2)	[−4, 0.66]	−33% (26%)	[−86%, 14%]	0.078	↓^ns^
Physical assault to service users	Lockdown 1	4.8	3.3 (1.7)	[−0.13, 6.7]	1.5 (1.7)	[−1.9, 5]	46% (52%)	[−56%, 150%]	0.188	↑^ns^
	Post-Lockdown 1	5	2.6 (1.4)	[−0.046, 5.4]	2.4 (1.4)	[−0.44, 5]	93% (53%)	[−17%, 194%]	**0.042**	↑^*^
	Lockdown 2	3.8	1.9 (1.5)	[−0.93, 5.2]	1.8 (1.5)	[−1.4, 4.7]	95% (78%)	[−72%, 243%]	0.112	↑^ns^
	Lockdown 3	5.7	1.9 (1.4)	[−0.75, 4.6]	3.8 (1.4)	[1.1, 6.4]	206% (74%)	[57%, 346%]	**0.006**	↑^*^
Non-physical assault to staff	Lockdown 1	8.6	13 (2.5)	[8.6, 18]	−4.8 (2.5)	[−9.8, 0.022]	−36% (18%)	[−73%, 0.16%]	0.**027**	↓^*^
	Post-Lockdown 1	12	7.8 (2)	[3.9, 12]	4.1 (2)	[0.038, 8]	52% (26%)	[0.49%, 102%]	**0.026**	↑^*^
	Lockdown 2	11	9.8 (2.2)	[5.7, 14]	1.1 (2.2)	[−3.5, 5.1]	11% (22%)	[−36%, 52%]	0.288	↑^ns^
	Lockdown 3	12	8.9 (2)	(7, 17)	2.8 (2)	[−1.3, 6.8]	32% (23%)	[−15%, 76%]	0.073	↑^ns^
Non-physical assault to service users	Lockdown 1	4.8	3.3 (1.7)	[0.15, 6.9]	1.5 (1.7)	[−2.1, 4.7]	46% (52%)	[−63%, 141%]	0.170	↑^ns^
	Post-Lockdown 1	5	2.6 (1.4)	[−0.022, 5.6]	2.4 (1.4)	[−0.62, 5]	93% (54%)	[−24%, 193%]	**0.049**	↑^*^
	Lockdown 2	3.8	1.9 (1.5)	[−1, 5.1]	1.8 (1.5)	[−1.3, 4.8]	95% (77%)	[−68%, 248%]	0.095	↑^ns^
	Lockdown 3	5.7	1.9 (1.4)	[−0.62, 4.7]	3.8 (1.4)	[0.98, 6.3]	206% (74%)	[53%, 339%]	**0.004**	↑^*^
Self-harm	Lockdown 1	2.8	4.3 (1.9)	[0.34, 8.2]	−1.4 (1.9)	[−5.4, 2.5]	−33% (44%)	[−126%, 59%]	0.219	↓^ns^
	Post-Lockdown 1	3.8	2.3 (1.5)	[−0.68, 5.5]	1.6 (1.5)	[−1.6, 4.5]	69% (66%)	[−73%, 199%]	0.148	↑^ns^
	Lockdown 2	3.8	1.3 (1.7)	[−1.7, 4.8]	2.4 (1.7)	[−0.98, 5.5]	182% (127%)	[−74%, 411%]	0.072	↑^ns^
	Lockdown 3	7.5	4.4 (1.5)	[1.6, 7.4]	3.1 (1.5)	[0.017, 5.9]	71% (34%)	[0.4%, 135%]	**0.026**	↑^*^
Damage to property	Lockdown 1	0.92	2 (0.8)	[0.37, 3.6]	−1 (0.8)	[−2.7, 0.55]	53% (41%)	[−138%, 28%]	0.103	↓^ns^
	Post-Lockdown 1	2.2	1.3 (0.65)	[0.07, 2.7]	0.85 (0.65)	[−0.56, 2.1]	65% (49%)	[−42%, 159%]	0.088	↑^ns^
	Lockdown 2	1.8	1.7 (0.68)	[0.44, 3.1]	0.14 (0.68)	[−1.2, 1.4]	8.1% (40%)	[−73%, 82%]	0.400	↑^ns^
	Lockdown 3	1.4	1.7 (0.63)	[0.53, 3]	−0.32 (0.63)	[−1.6, 0.85]	−19% (37%)	[−96%, 50%]	0.303	↓^ns^
**B. Restrictive interventions**									
Short-term seclusion hours	Lockdown 1	497	1168 (179)	[798, 1535]	−671 (179)	[−1038, −301]	−57% (15%)	[−89%, −26%]	**0.001**	↓^**^
	Post-Lockdown 1	722	877 (145)	[593, 1189]	−155 (145)	[−468, 128]	−18% (17%)	[−53%, 15%]	0.136	↓^ns^
	Lockdown 2	686	1018 (158)	[719, 1344]	−332 (158)	[−658, −33]	−33% (16%)	[−65%, −3.2%]	**0.021**	↓^*^
	Lockdown 3	426	726 (152)	[449, 1028]	−300 (152)	[−601, −23]	−41% (21%)	[−83%, −3.2%]	**0.020**	↓^*^
Long-term segregation hours	Lockdown 1	1551	647 (153)	[444, 856]	904 (104)	[695, 1107]	140% (16%)	[107%, 171%]	**0.001**	↑^**^
	Post-Lockdown 1	1478	571 (93)	[381, 752]	907 (93)	[727, 1097]	159% (16%)	[127%, 192%]	**0.001**	↑^**^
	Lockdown 2	1369	944 (108)	[725, 1155]	424 (108)	[214, 644]	45% (11%)	[23%, 68%]	**0.001**	↑^**^
	Lockdown 3	1645	867 (108)	[670, 1098]	779 (108)	[547, 976]	90% (12%)	[63%, 113%]	**0.001**	↑^**^
Enforced medication	Lockdown 1	0.92	1.2 (0.54)	[0.19, 2.3]	−0.3 (0.54)	[−1.4, 0.74]	−25% (44%)	[−144%, 60%]	0.301	↓^ns^
	Post-Lockdown 1	0.76	0.62 (0.44)	[−0.23, 1.5]	0.15 (0.44)	[−0.77, 0.99]	24% (71%)	[−124%, 161%]	0.350	↑^ns^
	Lockdown 2	0.62	1.1 (0.47)	[0.17, 2]	−0.45 (0.47)	[−1.4, 0.45]	−42% (44%)	[−133%, 42%]	0.159	↓^ns^
	Lockdown 3	2.5	0.59 (0.42)	[−0.23, 1.4]	1.9 (0.42)	[1, 2.7]	317% (71%)	[175%, 456%]	**0.001**	↑^**^
Continuous TESO hours	Lockdown 1	1749	1894 (217)	[1481, 2317]	−145 (217)	[−568, 269]	−7.7% (11%)	[−30%, 14%]	0.227	↑^ns^
	Post-Lockdown 1	1743	1402 (182)	[1045, 1790]	341 (182)	[−47, 698]	24% (13%)	[−3.4%, 50%]	**0.036**	↑^*^
	Lockdown 2	1163	1307 (205)	[893, 1716]	−143 (205)	[−553, 271]	−11% (16%)	[−42%, 21%]	0.227	↓^ns^
	Lockdown 3	1111	1845 (200)	[1434, 2283]	−734 (200)	[−1172, −323]	−40% (11%)	[−64%, −18%]	**0.003**	↓^*^
Intermittent TESO hours	Lockdown 1	3817	3229 (408)	[2441, 4036]	589 (408)	[−218, 1377]	18% (13%)	[−6.8%, 43%]	0.077	↑^ns^
	Post-Lockdown 1	2834	1852 (359)	[1183, 2599]	981 (359)	[235, 1651]	53% (19%)	[13%, 89%]	**0.008**	↑^*^
	Lockdown 2	4081	3251 (412)	[2249, 4075]	830 (412)	[5.3, 1632]	26% (13%)	[0.16%, 50%]	**0.026**	↑^*^
	Lockdown 3	4842	3070 (390)	[2300, 3888]	1772 (390)	[954, 2542]	58% (13%)	[31%, 83%]	**0.001**	↑^**^
**C. Care pathway progression**										
Admissions	Lockdown 1	0.54	1.6 (0.54)	[0.52, 2.8]	−1.1 (0.54)	[−2.2, 0.014]	−66% (34%)	[−138%, 0.86%]	**0.029**	↓^*^
	Post-Lockdown 1	1.1	1.5 (0.44)	[0.69, 2.4]	−0.42 (0.44)	[−1.3, 0.37]	−28% (30%)	[−90%, 25%]	0.169	↓^ns^
	Lockdown 2	1.3	1.9 (0.46)	[1, 2.9]	−0.58 (0.46)	[−1.6, 0.27]	−31% (24%)	[−82%, 14%]	0.103	↓^ns^
	Lockdown 3	1.2	1.2 (0.41)	[0.39, 2]	−0.0059 (0.41)	[−0.84, 0.76]	−0.51% (36%)	[−73%, 66%]	0.474	^−*ns*^
Discharges	Lockdown 1	1.2	1.9 (0.78)	[0.39, 3.6]	−0.72 (0.78)	[−2.4, 0.84]	−37% (40%)	[−122%, 43%]	0.172	↓^ns^
	Post-Lockdown 1	1.1	1.7 (0.62)	[0.52, 3]	−0.64 (0.62)	[−1.9, 0.54]	−38% (36%)	[−113%, 31%]	0.133	↓^ns^
	Lockdown 2	1.4	1.7 (0.66)	[0.41, 3.1]	−0.34 (0.66)	[−1.7, 0.98]	−20% (38%)	[−97%, 57%]	0.311	↓^ns^
	Lockdown 3	1.4	1.2 (0.62)	[0.082, 2.4]	0.22 (0.62)	[−1, 1.3]	19% (53%)	[−89%, 112%]	0.329	↑^ns^

*“Prediction” refers to the value expected based on data collected pre-pandemic, and actual refers to the observed value for each of the outcomes*.

*↑↓, direction of actual vs. prediction difference, with ↑ as actual greater than prediction and ↓ as actual less than prediction;*

* *p < 0.05;*

** *p ≤ 0.001; sd, standard deviation*.

#### Restrictive Interventions

Five categories were analyzed: STS hours, LTS hours, enforced medication, continuous TESO (Therapeutic Engagement and Supportive Observation) hours and intermittent TESO hours (see Table 1). Results showed a significant decrease in number of STS hours during Lockdown 1 (57%), Lockdown 2 (33%), and Lockdown 3 (41%) compared to what would have been expected based on prediction. In contrast, results showed a significant increase in the number of LTS hours at every time point during the pandemic; specifically, LTS hours recorded a 140% increase during Lockdown 1, 159% increase during post-Lockdown 1, 45% increase during Lockdown 2, and a 90% increase during Lockdown 3 (Table 2B). As for enforced medication, results indicated a significant increase (317%) during Lockdown 3 in comparison to prediction. Finally, the continuous and intermittent TESO results revealed a mixed pattern. Continuous TESO hours significantly increased (24%) post-Lockdown 1 but decreased (40%) during Lockdown 3. Intermittent TESO hours were significantly greater than prediction at post-Lockdown 1 (53%), Lockdown 2 (26%), and Lockdown 3 (58%) (Table 2B).

#### Care Pathway Progression

The models looking at care pathway outcome data, which included admissions and discharges, suggested an overall decrease across the pandemic. This included a significantly lower number of admissions (66%) during Lockdown 1 than would have been predicted based on pre-pandemic data (Table 2C).

### Changes in Service User Levels of Risk

#### HCR-20v3 Clinical and Risk Single Items Analysis

Item C5 (assessing problems with treatment or supervision response in terms of compliance and responsiveness) mean ranks significantly increased over time from pre-pandemic (mean rank 1.95) through to the first 6 months of the pandemic (mean rank 1.97) and into the second 6 months of the pandemic (mean rank 2.08), χ^2^(2) = 6.276, *p* = 0.043. *Post-hoc* comparisons revealed a significant difference between the first 6 (mean rank 1.46) and the second 6 months (mean rank 1.54), χ^2^(1) = 4.263, *p* = 0.039.

When ward level of security (medium and low) was explored, results showed a significant decrease in item C3 (assessing problems with symptoms of major mental disorders, including psychotic, mood, and other disorders) mean ranks across the 3 time points in service users in low security, χ^2^(2) = 8.222, *p* = 0.016, but not in medium security, χ^2^(2) = 0.442, *p* = 0.802. *Post-hoc* comparisons revealed a significant decrease only between the pre-pandemic period (mean rank 1.55) and the first 6 months (mean rank 1.45), χ^2^(1) = 4.0, *p* = 0.046.

Results also revealed that males (but not females) showed an increase in item C4 (which assesses affective, behavioral, and cognitive instability) mean ranks across the 3 time points, χ^2^(2) = 6.977, *p* = 0.031. *Post-hoc* comparisons revealed a significant increase in C4 score between pre-pandemic (mean rank 1.45) and the second 6 months (mean rank 1.55), χ^2^(1) = 3.846, *p* = 0.05. This significant increase in item C4 score was also observed between the first (mean rank 1.45) and second 6 months (mean rank 1.55), χ^2^(1) = 5.0, *p* = 0.025.

#### HCR20v3 Clinical and Risk Total Scores Analysis

Results revealed a significant main effect of gender on Clinical scale total score, F_(1, 121)_ = 6.003; *p* = 0.016. This suggests that female service users presented with significantly higher mean total scores (mean 6.21) on the Clinical scale than males (mean 4.703). Results also indicated a marginal interaction between gender and time point, *F*_(2, 121)_ = 2.877; *p* = 0.058. *Post-hoc* paired sample *t*-tests revealed that male service users had a significant increase in overall clinical risk between the first (mean 4.57) and the second 6 months (mean 4.91) of the pandemic, *t* (99) = 2.035; *p* = 0.045. Conversely, females did not show any significant changes across the 3 time points.

The same analysis was repeated using Risk scale total score. A significant main effect of gender was found, *F*_(1, 121)_ = 5.604; *p* = 0.019, with female service users (mean 5.81) scoring significantly higher than male service users (mean 4.72). No significant interaction between gender and time point was found.

## Discussion

The present study aimed to characterize the impact of the COVID-19 pandemic on service provision and implementation within a large inpatient forensic mental health service in West London. This was with the intention of informing decision-making should similar emergencies occur in the future. Our findings yielded several observations which deserve comment.

Like other health services in the UK, the COVID-19 pandemic considerably affected the implementation of therapeutic and social activities within the studied forensic service. The descriptive analysis of service delivery (Figure 2) found that a host of restrictions were implemented across the course of the pandemic following national guidance and coinciding with the three national lockdowns, with some only returning to “business as usual” relatively recently. Restrictions were made regarding in-person visitation for both professional and social contacts, with most visits taking place virtually rather than in-person. Section 17 Leave was restricted for a considerable period, and recreational and lifestyle activities were also restricted or unavailable for most of the pandemic. As stated above, each of these changes were carefully considered in regular COVID-19 governance meetings and implemented following government guidance but, together, these examples highlight how the pandemic markedly affected the day-to-day running of the service. It should, of course, be recognized that many of the service changes were implemented amid a fast-evolving situation in which there were rapid changes in national and NHS guidance regarding COVID-19.

Although these changes may have affected service user experience, they met their intention in that they were effective in managing COVID-19 transmission within the service: cases amongst service users remained mostly at low levels and staffing levels remained consistent throughout (Figure 3). It is also notable that, despite the considerable disruption caused to the service by the pandemic, admissions into the service were only affected during Lockdown 1 and discharge frequency remained comparable with pre-pandemic levels.

However, the service changes described above, alongside the broader impact of the pandemic across society, may have come with some costs to the service. Indeed, this study employed a series of causal impact models comparing actual vs. predicted values across a range of routine outcomes, namely incidents of violence, restrictive interventions, and care pathway progression. Before reviewing the findings, it is important to note that the routine outcome data are inherently linked to one another, and thus a reduction in one outcome may be linked to an increase in another, for example. Of course, although causal impact models aim to estimate causal effects, that is not to say that the COVID-19 related restrictions are a unitary explanation for changes in routine outcome; changes in outcome likely reflect more complex interactions between changes in practice and service user individual difference factors (such as symptomatology).

To the best of our knowledge, our study is the first empirical work directly assessing the impact of COVID-19 related restrictions on routine outcomes within a forensic mental health service. Therefore, our findings can only be discussed in relation to previous (pre-pandemic) work that has investigated service user experiences of restrictive interventions and how these relate to various clinical outcomes (24–27). As presented in Table 2, three of the indices of violence were significantly more frequent during Lockdown 3 (1st January 2021 to 31st March 2021) than would have been predicted based on pre-pandemic data–perhaps suggesting that the pandemic and implementation of pandemic-related restrictions during Lockdown 3 had a causal impact (increase) on incidents of violence. Studies conducted pre-pandemic indicate that perceived restrictiveness is negatively correlated with ward atmosphere (26) and that ward social climate predicts frequency of incidents of violence (19). Therefore, the present findings suggest that the implementation of pandemic-related restrictions might have exacerbated and made these effects even more severe, leading to an increase of incidents of violence. Indeed, for Lockdown 3, significant increases vs. prediction were observed in (i) physical assaults to service users, (ii) non-physical assaults to service users, (iii) incidents of self-harm. However, increases across these indices of violence are contrasted by an overall reduction in physical assaults by service users toward staff across the entire pandemic, reaching a statistically significant reduction vs. prediction during Lockdown 2. Taken together, these results suggest that forms of violence targeted specifically toward service users (including self) were significantly more frequent at Lockdown 3 than would have perhaps been predicted if the COVID-19 pandemic and its associated restrictions had not occurred. Pandemic-related restrictions (introduced particularly at Lockdowns 1 and 3) meant that service users spent more time mixing on-ward and in ward “bubbles”, rather than intermixing with other wards and accessing community leave as was the case prior to the pandemic. It could be that the observed increase in violence between service users is a result of this—perhaps due to incompatibilities or tensions festering on-ward across the course of the pandemic. The increase observed in incidents of self-harm vs. prediction (71%) during Lockdown 3 may also reflect this, as well as more general feelings of anxiety, frustration, and hopelessness in response to the pandemic. The significant reduction in incidents of physical assaults toward staff at Lockdown 2 is slightly more difficult to interpret, with general decreases in incidents of this type seen across the course of pandemic vs. prediction. One potential explanation could be that the pandemic reduced some of the factors that usually precipitate service user aggression toward staff. Service users may have recognized how staff were affected by the pandemic, perhaps increasing feelings of appreciation and sympathy, whilst reducing feelings of frustration, and thereby reducing frequency of violence toward staff.

Models revealed a 317% increase in use of enforced medication at Lockdown 3 in comparison to prediction. Typically, medication is enforced when prescribed medication is repeatedly refused and/or following a dramatic decline in mental state. The observed increase in use of enforced medication is consistent with the HCR-20v3 data, which revealed significantly higher scores in item C5 (problems with treatment or supervision response in terms of compliance and responsiveness) during the second 6 months of the pandemic (i.e., capturing Lockdown 3) than other pandemic time points. This could indicate that restrictions implemented during Lockdown 3 (along with the wider stresses of this stage of the pandemic) contributed to reduced willingness to comply with treatment and poorer symptomatic response to treatment, leading to a greater need for use of enforced medication. However, this is a tentative suggestion and should be substantiated by further research.

Similarly, a robust and highly significant causal effect of the pandemic on LTS hours was found. We observed an increase in the number of LTS hours at every time point during the pandemic compared to what would have been predicted by the pre-pandemic data. This increase in LTS hours is, in part, explained by the aforementioned policy change regarding converting from STS to LTS, but this policy change was implemented at limited intervals, namely during Lockdown 1, and during a short portion of Lockdown 3, and therefore cannot account for the significant increase in LTS hours recorded during the 16 weeks post-Lockdown 1 (1st June 2020 to 30th September 2020), 12 weeks during Lockdown 2 (1st October 2020 to 31st December 2020), and part of Lockdown 3 (1st January 2021 to 31st March 2021). As such, these results suggest an overall general increase in LTS hours across the whole pandemic. As with enforced medication, this increase in LTS hours may be a product of increased service user violence (and subsequent placement in LTS to manage risk) but could also reflect use of LTS as a risk management strategy, particularly in anticipation of COVID-19 transmission risks or issues with staffing. Pre-pandemic studies have found that patient perceptions of restraint were negatively associated with several clinical outcomes including hostility, depression, suicidal ideation, and psychological state (25). Therefore, any increases in perceived restrictiveness, including increases in restrictive interventions, might have contributed to an overall increase in poorer outcomes, such as the increased incidents of self-harm and violence between patients found in the present study.

In addition to analyzing routine outcomes via causal impact models, our single item HCR-20v3 analysis showed that service users in low security demonstrated more improvement in psychiatric symptomatology than those in medium security. This difference was particularly visible when comparing pre-pandemic ratings vs. ratings completed during the first 6 months of the pandemic. One interpretation is that service users in low security were less affected by the changes in service delivery and implementation than service users in medium security. The results also showed that male service users demonstrated more affective, behavioral, and cognitive instability during the pandemic in comparison to pre-pandemic, and thus perhaps suggesting that female service users were more resilient to service changes in response to the pandemic. Looking at changes in the total HCR-20v3 Clinical and Risk scales, we found that male service users (but not female service users) demonstrated increased overall clinical risk, in the second 6 months of the pandemic, which could contribute to the causal impact findings regarding increased incidents of violence and restrictive interventions during Lockdown 3.

### Recommendations and Practical Implications

This study has examined the potential effect of COVID-19 pandemic and its related restrictions on routine outcomes within an inpatient forensic service. It has described changes that were made in response to the pandemic and, using causal impact models, has identified that these changes, alongside the wider impact of the pandemic, may have contributed to a range of suboptimal outcomes during Lockdown 3, reflected in increased incidents of violence and increased use of some restrictive interventions. Thus, the findings presented here provide insight into how the COVID-19 pandemic may have affected forensic service implementation and outcome, and perhaps suggest that forensic services developing responses to the COVID-19 pandemic (or similar crises) should consider the following:

- The need for each ward to form a “bubble” during the pandemic meant that service users had less opportunity to mix with peers from outside their ward and engage in their usual off-ward activities and therapies. Whilst these restrictions mirrored the national experience in the wider community, it may be that the increased time spent confined with peers on-ward precipitated an increase service user-to-service user incidents of violence. If similar circumstances were to occur in the future, special attention may need to be paid to these dynamics, and perhaps trigger the development of on the ward interventions aiming at facilitating effective de-escalation [e.g., (28)].
- Incidents of violence may increase following repeated lockdowns, perhaps induced by changes in general symptomatology, reduced willingness to comply with treatment, and poorer responsiveness. Psychological work around resilience and/or coping with stress may be useful in mitigating this, along with working to minimize (where possible) the practical impact of lockdowns on the inpatient environment and procedures, whilst maintaining any required measures.
- Use of restrictive interventions, including seclusions and enforced medication, may increase during lockdown periods. Mental health providers should already have a restrictive intervention reduction programme or similar (29), as does the service under study, so it could be recommended that these programmes should have a particular focus on monitoring changes in restrictive interventions during a pandemic.
- Changes in symptomatology in response to the pandemic may vary depending on service user gender.
- More dynamic assessments of risk [e.g., Dynamic Assessment of Situational Aggression IV; (30)] could be useful if looking to monitor changes in risk more regularly than HCR-20v3. This might provide a more nuanced insight into the impact of pandemic-related restrictions and changes in service user risk.

### Limitations

There are some limitations within this study worth of note. One is the inference of causality regarding pandemic-related restrictions and corresponding changes in outcome. Whilst the causal impact models made predictions using weekly data collated for 2 years prior to the pandemic, it is difficult to say with certainty that the observed differences between prediction and actual outcome were due to the pandemic-related restrictions or the wider impact of the pandemic (e.g., worry about safety of loved ones). Similarly, the pandemic was organized as Lockdown 1, post-Lockdown 1, Lockdown 2, and Lockdown 3. Although this allowed us to compare routine outcomes across lockdown periods, this perhaps missed some of the more granular changes that occurred in the service in response to more specific government guidance (for example the movement into Tier 4 which occurred on 21st December 2020, affecting movement over the festive period). Furthermore, although Lockdowns 1 and 3 are presented as quite similar in terms of the types of restrictions that were implemented within the service, the subjective experiences associated with both lockdowns are likely to be quite different (e.g., feelings of anxiety, togetherness, confidence about the vaccination, extended periods without seeing family/friends in-person). It was, however, beyond the scope of this study to examine these qualitative aspects and their influence on the changes in routine outcomes observed here. As noted earlier, it is possible that service-user individual difference factors (e.g., symptomatology, age) contributed to the observed changes in routine outcome. It was, however, beyond the scope of this study to examine this descriptively or statistically due to lack of access to these data. This is an important consideration which should be addressed by future research examining COVID-19 related impacts within forensic services.

## Conclusion

This study has detailed a systematic analysis of the effect of the COVID-19 pandemic on service implementation and outcomes within a large inpatient forensic mental health service. Based on the presented findings, it appears that the pandemic had a negative impact on some aspects of service implementation (including significant changes to important therapeutic activities) and that the restrictions incurred by the pandemic may have negatively affected service outcomes (specifically increased incidents of violence and increase in some restrictive interventions) beyond what would have been expected had the pandemic not occurred. A list of recommendations for how to potentially apply these findings within service planning and implementation has been provided with the aim of informing future practice within inpatient forensic services.

## Data Availability Statement

The data analyzed in this study is subject to the following licenses/restrictions: Clinical routine data owned by West London NHS Trust. Requests to access these datasets should be directed to nicholas.stokes@westlondon.nhs.uk.

## Ethics Statement

The present study involved human participants and was therefore reviewed and approved by the Clinical Effectiveness and Audit Department (West London NHS Trust) and the Research Ethics Committee at Brunel University London–27418-LR-Dec/2020-29482-1. Written informed consent for participation was not required for this study in accordance with the national legislation and the institutional requirements.

## Author Contributions

IP, LA-W, NS, and VK contributed to the formulation of the research questions. NS and JR contributed to data gathering. IP and LA-W pre-processed and analyzed the data. IP, LA-W, NS, JR, and VK interpreted the results and drafted the manuscript, critically revised, and approved the version to be published. All authors contributed to the article and approved the submitted version.

## Conflict of Interest

The authors declare that the research was conducted in the absence of any commercial or financial relationships that could be construed as a potential conflict of interest.

## Publisher's Note

All claims expressed in this article are solely those of the authors and do not necessarily represent those of their affiliated organizations, or those of the publisher, the editors and the reviewers. Any product that may be evaluated in this article, or claim that may be made by its manufacturer, is not guaranteed or endorsed by the publisher.
